# Antibiotic-Loaded Polymethylmethacrylate Beads and Spacers in Treatment of Orthopedic Infections and the Role of Biofilm Formation

**DOI:** 10.3389/fmicb.2019.01626

**Published:** 2019-07-25

**Authors:** Tom A. G. van Vugt, Jacobus J. Arts, Jan A. P. Geurts

**Affiliations:** Maastricht University Medical Centre (MUMC+), Maastricht, Netherlands

**Keywords:** prosthetic join infection, polymethylmethacrylate, osteomyelitis, antibiotic loaded bone cement, gentamicin beads, antibiotic loaded acrylic cement spacer

## Abstract

Polymethylmethacrylate (PMMA) also referred as (acrylic) bone cement is a non-degradable biomaterial that has been used in clinical orthopedic practice for several decades. PMMA can be used in a plain formulation, but is often used in an antibiotic-loaded formulation in (primary and revision) arthroplasty and in treatment of orthopedic infections as prosthetic joint infections (PJI) and chronic osteomyelitis. In treatment of PJIs antibiotic-loaded PMMA is often used as a carrier material for local antibiotic delivery in addition to treatment with systemic antibiotics. In this case, the antibiotic-loaded PMMA is often used as a spacer or as a bead chain. Since the introduction of PMMA as an antibiotic carrier there is a tremendous amount of scientific and clinical papers published, which studied numerous different aspects of antibiotic-loaded PMMA. This paper will review the research regarding basic principles of antibiotic-loaded PMMA as mechanism of action, antibiotic-release capacities, choice of antibiotics and influences on mechanical properties of PMMA. Subsequently, concerns regarding the application of antibiotic-loaded PMMA, biofilm formation, antibiotic resistance and local or systemic toxicity will be discussed. In addition to these subjects, the role of antibiotic loaded PMMA in clinical treatment of PJIs and chronic osteomyelitis is discussed in the final part of this paper.

## Introduction

Bacterial infections are one of the most devastating complications in the field of orthopedic surgery. Especially infections related to implants e.g., cases of infection in total joint arthroplasty (TJA), or infections related orthopedic traumatology where implants are used for bone fixation are burdensome and difficult to treat. The infection rates vary based on the etiology of the infections, this review highlights the orthopedic prosthetic joint infections (PJIs) and trauma related chronic osteomyelitis. In cases of PJI the infection rates vary from <1% in primary total hip arthroplasty up to 10 to 14% in spine surgery or revision arthroplasty (Phillips et al., 2006; Olsen et al., 2008; Parvizi et al., 2008a). In the specific case of orthopedic traumatology, the chronic osteomyelitis rates are even up to 50% according studies regarding surgical fixation of open fractures (Gustilo et al., 1990; Trampuz and Zimmerli, 2006).

The number of orthopedic implant surgeries is rising in the past years and is expected to increase in the upcoming years. Not solely the absolute numbers of PJIs are increasing, but also the relative infection rates are increasing (Dale et al., 2009). These numbers are rising due to the aging population and a higher demand for joint replacements due to higher activity levels of these patients. Due to these facts, the average patient undergoing TJA has more comorbidities resulting in a higher risk of infection (O’Toole et al., 2016). Treatment of orthopedic infections as PJIs and chronic osteomyelitis are difficult, invasive, expensive, and they cause a significant increase of morbidity and even mortality (Zmistowski et al., 2013). Treatment of PJIs or chronic osteomyelitis differs based on their etiology, but the common denominator for treatment is multimodal; containing surgical debridement, systemic antibiotic treatment and local antibiotic treatment. PJIs can be treated by debridement surgery, antibiotics, irrigation and implant retention (DAIR), a one-stage replacement surgery, or a two stage replacement surgery depending on the duration of infection, the type of causative pathogen, status of soft tissues and the health status of the patient (Zimmerli et al., 2004). Chronic osteomyelitis can be treated in a one-stage or two-stage surgery as well and the choice of treatment is based on the same conditions. Two-stage surgeries are required in delayed infections, severe infections with systemic symptoms, infections with pathogens that are difficult to treat, and cases of infection with compromised soft tissues (Lazzarini et al., 2004). The first stage of this treatment algorithm consists of implant or hardware removal, debridement surgery and implantation of a local antibiotic carrier. During the second stage, the local antibiotic carrier is removed. Subsequently, in the case of PJIs, a new endoprosthesis is implanted, where in the case of chronic osteomyelitis the bone defect is filled with either allograft, autograft or a bone void filling biomaterial.

As mentioned before, local antibiotic therapy is important in treatment of PJIs and chronic osteomyelitis. For the antibacterial effectiveness, it is important that the local antibiotic concentration exceed the minimal inhibitory concentrations (MIC) of the causative pathogens (Jacobs, 2001). Depending on choice of antibiotics, it is important to exceed the MIC for a longer period or to achieve the highest concentrations, because antibiotics have different activity patterns. Some antibiotics act based on time depending killing principles where the duration of exposure above MIC is very important (macrolides, β-lactam antibiotics, and clindamycin). Other antibiotics are concentration dependent where the highest possible local concentration above MIC should be achieved (aminoglycosides, quinolones, and vancomycin) (Jacobs, 2001). Polymethylmethacrylate (PMMA), also called bone cement or acrylic bone cement, is a widely accepted carrier material for this local antibiotic delivery and is able to exceed the required MIC. This so-called antibiotic-loaded PMMA or antibiotic loaded bone cement (ALBC) is applied in different forms, but is generally applied as a bead (or bead chain) or a spacer. This paper will highlight basic principles of antibiotic-loaded PMMA in treatment of PJIs and chronic osteomyelitis and its effect on biofilm formation.

## History and Development of Antibiotic Loaded PMMA

Bone cement is used for almost 150 years since Gluck introduced it in 1870 for fixation of a total knee prosthesis (Brand et al., 2011). Back then the bone cement consisted mainly of plaster and colophony, but since the introduction of this bone cement many researchers were looking for an optimization for fixating implants to bone. In 1960, Sir John Charnley introduced PMMA as a new type of bone cement for fixation of the total hip prosthesis in total hip replacement surgery and since this introduction, PMMA is used as golden standard in fixation of cemented total joint arthroplasty (Charnley, 1960; Charnley, 2010). Only a few years after PMMA was introduced, Buchholz and Engelbrecht introduced the concept of combining PMMA and antibiotics in order to achieve a high local antibiotic concentration in treatment of bone infection (Buchholz and Gartmann, 1972). With his research, Buchholz showed that PMMA bone cement was capable of releasing different materials or substances like antibiotics and cupper ions from its surface. At first, antibiotic-loaded PMMA was used to prevent from bacterial infections, but in 1972, Buchholz introduced this principle as a treatment strategy for PJIs.

In the past decades, the optimization of antibiotic-loaded PMMA became an important research topic in the improvement of prophylaxis and treatment of PJIs (Walenkamp, 2007). PMMA is fabricated by mixing a polymer powder with a monomer liquid resulting in an exothermic polymerization reaction leading to a solid rigid material. Antibiotics and other substances are added to PMMA by admixing them to the polymer powder before adding the monomer. As a result, the antibiotics are incorporated between the PMMA chains during the polymerization process (Walenkamp et al., 1986). Antibiotic release after incorporation is based on reciprocal diffusion and is divided into two different phases. The Initial release of antibiotics, called burst release, is a quick response after implantation resulting in an early (minutes to hours) high local concentration of antibiotics. This burst release is a surface phenomenon where the antibiotics of the surface dissolute into the body fluids out of the PMMA. The second phase, called sustained release, follows after several days and results in a significantly lower, but prolonged local antibiotic concentration. Sustained release is a phenomenon where water-soluble antibiotics diffuse out of the PMMA after depth penetration of the water containing body fluids; because PMMA is hydrophilic it will attract water molecules resulting in a release of the water-soluble antibiotics into the body fluids.

Different *in vitro* studies showed that the pharmacokinetic release profiles of PMMA can be optimized by adjusting several properties of the PMMA. Since antibiotics are released by dissolution after contact with body fluids, an increase of the surface roughness and the porosity of the PMMA result in an increase of surface area, leading to an increased antibiotic release (van de Belt et al., 2000b). The increase of porosity of PMMA can be easily achieved by hand mixing the PMMA instead of vacuum mixing (van de Belt et al., 2000a). Another possibility to increase the release capacities of the PMMA is to increase the dissolution capacity by adding polymeric fillers (e.g., xylitol and glycine) and using highly water-soluble substances (Rasyid et al., 2009).

## Choice of Antibiotics

It is important to select the type of antibiotics with caution, since not all types of antibiotics are suitable for incorporation in PMMA, see Table 1. Because antibiotics are incorporated between the PMMA chains, it is important to realize that different types of antibiotics can have different effects on the PMMA. Some antibiotics can influence the orientation and the cross-linking of the polymer chains resulting in changes in the viscosity after mixing, the polymerization time, concentration of incorporated antibiotics, and the mechanical strength and stiffness or of the PMMA. In addition to the effects of antibiotics on PMMA, PMMA can influence the effectiveness of antibiotics as well. Antibiotics have to be heat stable due to the exothermic polymerization reaction; antibiotics must be water soluble for dissolution out of the PMMA and the must be available in powder form for admixing them into the polymer powder. Besides, these physicochemical material properties the antibacterial properties are of great importance for treatment of orthopedic infections. Ideally, the selected antibiotics should be bactericidal, should be broad-spectrum and they should have a low risk of resistance induction, hypersensitivity and/or allergies. Antibiotics often used in clinical practice are gentamycin and vancomycin in Europe, and Tobramycin in the United States.

**TABLE 1:** Overview of common used antibiotics and their suitability for incorporation in PMMA.

**Type of antibiotics**	**Suitability and remarks**	**Spectrum**
Gentamicin	Good; most common used in Europe	Gram neg., *E. coli*, Klebsiella, some pseudomonas and aerobic
Vancomycin	Good; especially in combination with gentamicin	Gram pos.; including MRSA and MRSE
Tobramycin	Good; common used in United States	Gram neg.; especially pseudomonas
Clindamycin	Good; especially in combination with gentamicin	Gram pos., Anaerobes
Erythromycin	Good	Aerobic gram pos. cocci, bacilli
Ciprofloxacin	Good	Gram neg; including enterobacteria
Cephalosporin’s,	Moderate; short acting due to hydrolisis and not heat stable	Depending on generation: basically 1st and 2nd gram pos., 3rd and 4th Gram neg.
Tetracycline,	Poor; not heat stable and high risk of resistance	Gram pos.; Gram neg.
Rifampicin	Poor; decreases mechanical properties of PMMA	Biofilm activity against *S. aureus* biofilm

Depending on the clinical situation, it is also possible to add multiple antibiotics to PMMA instead of adding one single type of antibiotics. These so-called, double-antibiotic bone cements are often used in cases of septic revision arthroplasty after PJIs for implant fixation (Frommelt and Kuhn, 2005). The rationale for using PMMA bone cements with two different types of antibiotics is based on the broadening of the antibiotic spectrum, but is also based on the synergistic effects of some combinations of antibiotics (Sanz-Ruiz et al., 2018b). The synergistic effects of the combination of these antibiotics cause a mutual increase of antibiotic release but can also increase the mutual antibacterial efficacy. Good combinations for double-antibiotic bone cements are listed in Table 2.

**TABLE 2:** Common used combinations of antibiotics for double-antibiotic bone cements.

**Antibiotics**	**Spectrum**	**Effects**
Gentamicin + Vancomycin	Covering almost all pathogens (incl. MRSA and MRSE)	Synergistic in amount of antibiotic release
Gentamicin + Clindamycin	Broad spectrum except some pathogens as streptococcus spec.	Synergistic in antibacterial effects and antibiotic release

*MRSA = methicillin resistant *Staphylococcus aureus*, MRSE = methicillin resistant *Staphylococcus epidermidis*.*

## Application of Antibiotic-Loaded PMMA

In the current orthopedic practice, antibiotic-loaded PMMA is used as a prophylactic strategy to prevent from PJIs in total joint arthroplasty, but antibiotic-loaded PMMA is also used in treatment of orthopedic infections as PJIs and chronic osteomyelitis. Different studies have shown that antibiotic-loaded PMMA can be used in the vast majority of the cases of primary and revision arthroplasty for implant fixation resulting in lower septic revisions and longer prosthesis survival (Engesaeter et al., 2003; Jamsen et al., 2009). Although these results, there is opposing data that states there is no reduction in PJIs when using antibiotic-loaded PMMA (Hinarejos et al., 2013). Due to these opposing data there is no globally consensus in using the antibiotic-loaded PMMA. In Scandinavia and Western-Europe antibiotic-loaded PMMA is commonly used in primary and revision arthroplasty, where in the United States this antibiotic-loaded PMMA is only approved for revision arthroplasty. As discussed, antibiotic-loaded PMMA is suitable for different clinical situations, there are some major differences in the cement used. Antibiotic-loaded PMMA used in infection prophylaxis is only used as bone cement for the fixation of endoprosthesis and has significantly lower concentrations, because otherwise the biomechanical strength is decreased (Lautenschlager et al., 1976a, b), resulting in PMMA not suitable for weight bearing and proper fixation of the implants. Exceptions are the so-called revision cements, which are double-antibiotic bone cements and/or have higher antibiotic concentrations compared to antibiotic-loaded PMMA suitable for prophylaxis (Frommelt and Kuhn, 2005; Fink et al., 2011b). These are commercially available cements with specific combinations of predefined antibiotics, used for fixation of implants after septic revisions in PJI treatment. These specific antibiotic combinations do have good biomechanical properties for implant fixation as well.

**FIGURE 1:**
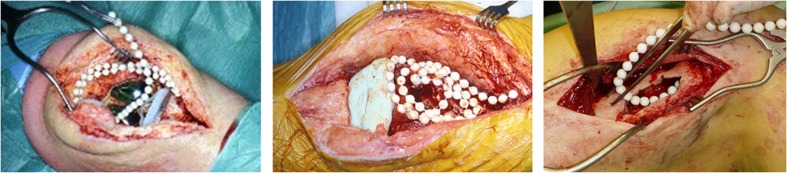
Examples of the application of antibiotic loaded Polymethylmethacrylate in the orthopedic practice. In figure on the left side, solely antibiotic loaded PMMA-beads are used in treatment of infected TKP. In the middle beads and a (hand-molded) spacer are used for treatment of an infected TKP. In the figure on the right side, PMMA-beads are used in treatment of chronic osteomyelitis.

In treatment chronic osteomyelitis or PJIs after removing the endoprosthesis, antibiotic-loaded PMMA is applied as bead or bead chain but also as a spacer, see Figure 1 and Table 3. Since these beads or spacers are a temporary solution, biomechanical requirements are of less importance since they are not used for definitive implant fixation and long-term weight bearing; therefore the antibiotic concentrations are higher. In these cases, treatment of infection is based on proper dead space management, soft tissue management and local antimicrobial therapy. Good dead space management achieves high antibiotic concentrations by filling the bony defect after surgery as much as possible to reduce the amount of hematoma, since there is less volume of blood/hematoma to dissolve the total amount of antibiotics. This results in higher amount of tissue penetration of the local antibiotics.

**TABLE 3:** Advantages and disadvantages of antibiotic loaded PMMA beads and spacer.

**Beads or bead-chains**		**Spacers**	
**Advantages**	**Disadvantages**	**Advantages**	**Disadvantages**
Higher antibiotic concentrations	Impaired local anatomy	Preservation of local anatomy	Relatively low antibiotic concentrations and short period
Relatively inexpensive	Extensive scar tissue	Pre-fabricated spacers are expensive	Dislocation/migration of spacers
Low complication rates		(Possible) joint mobility (and weight baring)	Fractures of spacers
Easy to use, implant and remove			Spacer fractures
Longer period MIC			

**FIGURE 2:**
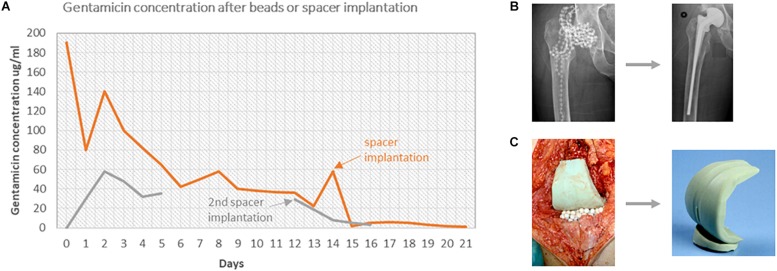
**(A)** The gentamicin concentrations in exudate are shown over time in two different cases. Patient A had an infected THP, treated with 300 gentamicin beads at first surgery and a spacer at the second surgery. Patient B had an infected TKP, treated with 60 beads and 1 spacer at the first surgery and 2 spacers after the second surgery. **(B)** X-ray images of treatment of patient A. **(C)** Images of the treatment of patient B.

PMMA beads enable the possibility to fill the entire infected cavity with beads to reduce the dead space and achieve a higher surface area for antibiotic release compared to a solid PMMA plug (Klemm, 1979; Walenkamp, 2007). This also leads to a reduced amount of hematoma, which results in a higher local antibiotic concentration. After implantation PMMA beads reach their maximum concentration in the first 2–3 days after surgery leading to exudate concentrations of about 300–400 μg/ml, depending on the amount of implanted beads (Walenkamp, 2007) (Figure 2). The amount of beads implanted is limited by the size of the defect in cases of chronic osteomyelitis but in PJIs of total knee and total hip arthroplasty, respectively 180 to 360 beads can be implanted after removal of the prosthesis (Walenkamp and van Rens, 1982). In order to achieve even higher antibiotic concentrations or to fill smaller cavities, *in vitro* and *in vivo* studies showed that smaller PMMA beads could be used. These so-called mini-beads (3 × 5 mm instead of 7 × 7 mm) release up to 93% of the added antibiotics (vs. 24% in normal PMMA beads) and achieve antibiotic concentrations up to seven times higher compared to normal PMMA beads (Walenkamp, 1989). These mini-beads have a similar antibiotic release period with sufficient release concentrations in comparison to the normal PMMA beads (Walenkamp, 1989; Klemm, 2001). Both normal and mini-beads should be removed after several weeks since the release concentrations are decreasing and might drop below the MIC and can subsequently form a local substratum for bacterial inoculation (Walenkamp et al., 1986; Nelson et al., 1992).

**FIGURE 3:**
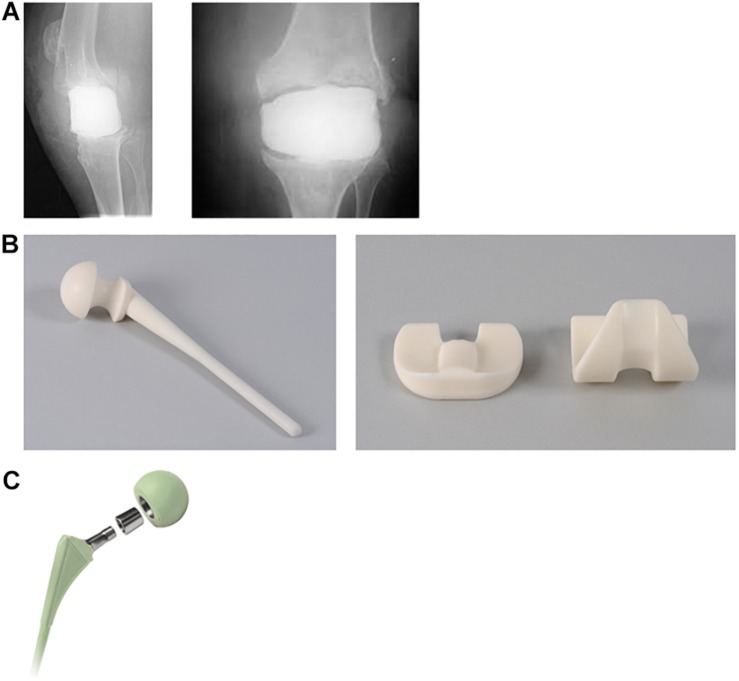
Different generations of hip and knee spacers. **(A)** Hand molded knee spacer (first generation). **(B)** Spacer made with pre-fabricated molds for hip and knee (second generation) (Tecres, Italy is the owner of the copyrights and all other intellectual property rights in relation to this picture). **(C)** Mobile bearing spacer (third generation) (Biomet Stage One Select system; Zimmer Biomet is the owner of the copyrights and all other intellectual property rights in relation to this picture).

Antibiotic-loaded PMMA spacers are frequently implanted during the first stage of two-stage treatment of PJIs after removal of the prosthesis. Implantation of the antibiotic-loaded cement spacer results in local antibiotic release, preservation of anatomical structures, decreased scar-tissue formation (arthrofibrosis) and (partial) joint mobility, which all favor the re-implantation of the definitive prosthesis during the second stage of PJI treatment, see Table 3. Different studies showed that the antibiotic release concentrations of the PMMA spacers exceed MICs of most pathogens in the first few hours to days of implantation (Bertazzoni Minelli et al., 2004; Balato et al., 2015). However, there are only a few studies concerning the prolonged *in vivo* antibiotic release concentrations and the current evidence regarding these concentrations is contradictory and inconclusive. Some studies suggest that antibiotic release concentrations remain above MIC for several months (Masri et al., 1998; Hsieh et al., 2006; Fink et al., 2011b). In contrast to these studies, there are studies, which show that release concentrations drops below MIC after 7 to 14 days (Kelm et al., 2006; Anagnostakos et al., 2009). In comparison to antibiotic-loaded PMMA beads, spacers have a relatively low maximum antibiotic concentration and their antibiotic release time above MIC is short (Henry and Galloway, 1995; Greene et al., 1998; Moojen et al., 2008). This is the result of the smaller surface area of the spacers. Since the introduction of antibiotic-loaded cement spacers, the biomechanical properties of these spacers have been developed extensively. First-generation spacers were mono-block, hand-molded spacers. These spacers could be adjusted to the patient specific anatomy, antibiotics can be incorporated selective, are easy to handle and relatively inexpensive. Although these advantages, first generation spacers are static and disable any articular motion resulting in higher fracture risks, bone loss, and extension mechanism erosion (Leunig et al., 1998; Hsieh et al., 2005; Jung et al., 2009; Struelens et al., 2013). Second-generation spacers are hand-made spacers as well, but are made with help of pre-fabricated molds provided by the industry. In addition, these spacers were often reinforced leading to a lower risk of spacer failure, but since these spacers were still static, the risks for the other complications remained. Since the introduction of the third-generation antibiotic-loaded PMMA spacers a complete new type, so called mobile or articulating spacers, which enable higher mobility of patients and reduced the risks of arthrofibrosis, see Figure 3. Examples are for instance the StageOne^TM^ Select System (Zimmer Biomet Inc., Warsaw, IN) and the PROSTALAC^®^ spacer (DePuy Orthopedics Inc., Warsaw, IN). Some studies suggests that mobile spacers might even result in better infection treatment, although this is still under debate due to the absence of unequivocal evidence (Chiang et al., 2011; Romano et al., 2012a, b; Voleti et al., 2013; Gehrke and Haasper, 2014). Disadvantage of these mobile spacers are the possible release of debris by eroding PMMA particles (Fink et al., 2011a) and associated wear particle induced osteolysis.

## Biofilms

Reduction of biofilms or prevention/inhibition of biofilm formation remains one of the major challenges in prevention and treatment of PJIs and chronic osteomyelitis. The phenomenon of biofilm formation is often termed “the race for the surface,” which was first described by Gristina (1987) and refers to the challenge between eukaryotic cells and bacteria to attach to the foreign body material e.g., the orthopedic implant (Gristina, 1987; Gristina et al., 1988). When bacteria adhere to the surface of the implant, the entire process of biofilm formation starts with encapsulating itself for protection to the human immune system, resulting in a mature biofilm filled with bacteria that will detach and disperse into the surrounding tissues and blood (Arciola et al., 2012).

Since bacteria in biofilm are protected by the biofilm layer they cause clinically relevant therapeutic and diagnostics challenges. The biofilm may cause difficulties in the microbiological diagnosis because the biofilm matrix can impede culturing of the bacteria, which can lead to false-negative cultures and missed diagnosis of infections. In order to prevent this there are many new developments in detaching the bacteria from their biofilm for better diagnosis (Dibartola et al., 2017).

Therapeutic difficulties caused by biofilms are related to the decreased susceptibility for (local and systemic) antibiotics. The extracellular matrix of the biofilm causes an additional barrier resulting in decreased diffusion. In addition, substances of the matrix react with the antibiotics and thereby decrease the transport rates (Donlan and Costerton, 2002). Besides the protection by the biofilm matrix, the bacteria inside the biofilm have a decreased cell metabolism, resulting in lower cell growth/division and are thereby less sensitive for antibiotics (Duguid et al., 1992). Due to these protection mechanisms antibiotic concentrations must far exceed the normal MIC (Stewart and Costerton, 2001). Although this is a widely accepted clinical opinion, literature regarding the effects of these high local antibiotics concentrations on bacterial biofilms is not convincing neither conclusive (van de Belt et al., 2000a; Jiranek et al., 2006; Griffin et al., 2012). Thereby it is know that (plain) PMMA is a biomaterial on which bacterial adhere best and can be colonized easily if antibiotic concentrations are insufficient (Bertazzoni Minelli et al., 2011). The anti-biofilm effects depend on the capabilities of bacteria to survive in high local antibiotic concentrations and their ability to adhere onto the surface of implants and subsequently form a biofilm. These characteristics depend among others, of the type of bacteria, the susceptibility of bacteria, the type and amount of antibiotics used. For example, when treating biofilm related infections, rifampicin is known to have a good effect of staphylococcal biofilm breakdown and killing bacteria in their stationary phase (Sendi and Zimmerli, 2012; Zimmerli, 2014; Zimmerli and Sendi, 2017). However, admixing rifampicin into PMMA interferes with the polymerization resulting in reduced mechanical properties (Sanchez et al., 2015; Shiels et al., 2017).

Different *in vitro* studies showed that high local antibiotic concentrations inhibit, but not fully avoid, biofilm formation (Bertazzoni Minelli et al., 2011; Balato et al., 2018). These studies showed that the formed biofilm on the antibiotic loaded cement was less thick in comparison to the plain cements, but there still was bacterial adhesion and biofilm formation. The combination of antibiotics as gentamicin and clindamycin or gentamicin and fusidic acid are more effective in preventing biofilm formation but cannot fully prevent from bacterial adhesion and biofilm formation (Neut et al., 2005; Ensing et al., 2008). Although this persisting biofilm formation on antibiotic loaded PMMA implants there is still a little clinical data regarding the clinical relevance of these studies. Some *in vivo* studies showed antibiotic-loaded PMMA beads and spacers colonized with bacterial biofilms but not all of these clinical cases showed clinical signs infection or positive cultures in the periprosthetic tissues (Neut et al., 2001; Anagnostakos et al., 2008). This confirms the complexity and the large amount of variable factors (selection of antibiotics, type of cement; duration of therapy, etc.) on which the treatment of biofilm related infections depends. Therefore, it is important that additional studies regarding prevention of biofilms; new treatment principles of biofilms; and clinical relevance of biofilms on temporary implants as beads of spacers should be performed.

## Toxicity and Resistance

One of the major concerns in administration of (local) antibiotics are the toxic side effects, especially cytotoxicity and systemic toxicity. Cytotoxicity after local antibiotic therapy is studied on cell survival and the capacity to recover of eukaryotic cells (like osteoblasts) in different *in vitro* studies. These studies showed good cell survival/recovery capacities after high antibiotic concentration exposure for antibiotics as tobramycin, vancomycin and gentamicin (Miclau et al., 1995; Edin et al., 1996; Isefuku et al., 2003). For most antibiotics, the tested toxic concentrations exceeded the maximum observed local *in vivo* antibiotic concentration. Although the application of local antibiotics seems safe, *in vivo* human studies to confirm these data are scarce. Systemic side effects caused by high systemic antibiotic concentrations are rare, but enhance especially nephrotoxicity, hepatotoxicity, and ototoxicity in application of gentamycin or vancomycin. Multiple pharmacokinetic studies regarding antibiotic concentrations showed serum and urine concentrations below toxic thresholds for specific antibiotics (Salvati et al., 1986; Walenkamp et al., 1986; Springer et al., 2004; Hsieh et al., 2009). Although, a meta-analysis of Luu et al. (2013) described some cases of nephrotoxicity, acute renal failure, for high-dose antibiotic loaded PMMA. To our knowledge, there is no clinical evidence of a relation between the application of low-dose antibiotic loaded PMMA and any local or systemic toxicity.

Another major concern in the application of antibiotic-loaded PMMA is the increase of bacterial antibiotic resistance. It is thought that after release of the majority of the antibiotics out of the antibiotic-loaded PMMA, the remaining antibiotics lead to sub-inhibitory concentrations, causing mutational antibiotic resistance (Hope et al., 1989; Thomes et al., 2002). In addition, it is know that plain PMMA has an optimal surface of colonization and when the antibiotic release of antibiotic-loaded PMMA drops below MIC the implanted PMMA becomes a local substratum for bacterial inoculation (Kendall et al., 1996; Neut et al., 2001). Although these concerns there is no clinical evidence that using antibiotic loaded PMMA in prophylaxis or treatment of PJIs and chronic osteomyelitis leads to an increase of antibiotic resistance. In addition, a study from the United States showed that there was no increase in antibiotic resistance after switching from plain PMMA to antibiotic loaded PMMA in TKA (Hansen et al., 2014).

## Clinical Results

With the introduction of antibiotic-loaded PMMA around the 1970s and the specific gentamicin-loaded PMMA a few years later, the prevalence of PJIs decreased from around 5% to less than 1% (Buchholz, 1979; Rottger et al., 1979). After successful application of antibiotic loaded PMMA in prophylaxis in TJA, different surgeons started to use gentamicin-loaded PMMA for fixation of the prosthesis in revision surgery. To our knowledge, there are only a few randomized controlled trials (RCTs) evaluating the effectiveness of applying antibiotic-loaded PMMA over plain PMMA in infection prophylaxis (Josefsson et al., 1981; Chiu et al., 2002). These studies showed a significant decrease in infection rates after application of antibiotic loaded PMMA for fixation of the total hip or total knee prosthesis. A meta-analysis by Parvizi et al. (2008b) regarding the risk reduction of infection when using antibiotic-loaded PMMA vs. plain PMMA for THA showed infection rates of 1.2% vs. 2.3% respectively, leading to a risk reduction of almost 50% (Parvizi et al., 2008b). This data is substantiated by some large retrospective studies of the Norwegian Arthroplasty register and the Swedish Joint Registration (Malchau et al., 1993; Engesaeter et al., 2003). The data of the Norwegian register showed an increased infection risks of 1.8 in using plain cement over antibiotic-loaded cement, where the Swedish registry data showed that the effectiveness of the application of antibiotic-loaded PMMA seemed to even higher for revision arthroplasty compared to primary joint arthroplasty. In addition to these data a study from the Finnish knee register showed that revision surgery for all reasons decreased (Jamsen et al., 2009). This was confirmed by a French study regarding total hip arthroplasty, where also was shown that the general survival of a total hip prosthesis cemented with antibiotic-loaded PMMA was higher in comparison to the uncemented total hip prosthesis (Colas et al., 2015). Although these results seems to be convincing there are a few studies suggesting that fixation with antibiotic-loaded cements in primary arthroplasty is not beneficial to fixation of with plain cements (Hinarejos et al., 2013, 2015). These studies state that antibiotic loaded cements should be used in specific situation with patients with high risks for infection, because of the risks of toxicity, hypersensitivity and possible antibiotic resistance in combination with the higher costs might outweigh the inconclusive evidence of infection reduction. These conflicting studies result in an absence of a global consensus strategy for using antibiotic loaded cements in primary arthroplasty and the usage of antibiotic loaded cement in primary arthroplasty is now mostly based on local guidelines and surgeons’ preferences.

Antibiotic loaded PMMA spacers are used over two decades in two-stage PJI treatment with good clinical results. Different studies show that in treatment of infected THA as well in treatment of TKA, eradication rates of two-stage treatment are successful in 85 to 100% (Haddad et al., 2000; Hsieh et al., 2005; Citak et al., 2014). However, there is still debate about several aspects of this two-stage treatment, e.g., spacer implantation period, duration of systemic antibiotic period, choice of mobile vs. static spacers and the type of prosthesis used in re-implantation. The period of spacer implantation and the duration of systemic antibiotics are varying from days to years, but in most studies, this is depending on clinical and biochemical parameters resulting in an average implantation period of 6 to 12 weeks (Masri et al., 1998; Hsieh et al., 2005). This highlights the importance of a proper surgical debridement during this two-stage treatment of PJIs. Although it seems obvious that a re-implantation of a cemented prosthesis with antibiotic-loaded PMMA should be advantageous over re-implantation of an uncemented prosthesis, high quality clinical evidence is scarce (Parvizi et al., 2008b).

Klemm (1979) first introduced antibiotic-loaded PMMA beads for treatment of chronic osteomyelitis in 1979 instead of larger pieces of hand-molded antibiotic PMMA spacer-like plugs. The PMMA beads were loaded with gentamicin and were applied as bead chains, because these bead chains achieved higher local antibiotic concentrations. His first study showed good result with a success rate of eradication of infection 91.4% in 128 cases. After publication of this study, only two low quality RCTs have been performed. The study of Blaha et al. (1993) compared two-stage treatment with antibiotic-loaded PMMA beads with or without additional systemic antibiotics and did not find a significant difference (Blaha et al., 1993). Shih et al. (2005) compared the effectiveness of a two-stage surgery with antibiotic-loaded PMMA implantation over one-stage debridement with solely intravenous antibiotics where for both groups a success rate of 100% was observed (Shih et al., 2005). In addition to these RCTs, there are several large observational studies with eradication rates of 87–100% for this two-stage treatment algorithm with antibiotic-loaded PMMA bead implantation (Majid et al., 1985; Jerosch et al., 1995; Walenkamp et al., 1998). Although the evidence regarding the superiority of using antibiotic-loaded PMMA needs to be substantiated, this two-stage treatment principle is considered as the golden standard in treatment of chronic osteomyelitis.

## Future Perspectives

As PJIs and chronic osteomyelitis remain a substantial problem in orthopedic surgery, improvement of treatment of PJIs and chronic osteomyelitis with local antibiotic-loaded PMMA continues. Current research concepts are regarding surface modification and microencapsulation of specific antibiotics as e.g., rifampicin to enable biofilm breakdown and infection treatment by rifampicin loaded PMMA cement with good mechanical properties (Sanz-Ruiz et al., 2018a).

In addition to development of antibiotic-loaded PMMA in prevention and treatment of PJIs, new carriers and coatings for controlled release of local antibacterial agents are important research topics for biofilm infections. Thereby, new carriers are interesting prophylactic opportunities for the uncemented prosthesis in TJA as well. These new strategies are based on adaptation of the surface of implants to prevent from infection, basic principles are (1) passive surface modification, (2) active surface modification and (3) local carriers/coatings, see Table 4 (Romano et al., 2015). Examples of new surface modifications are e.g., antibiotic releasing biodegradable polymers, antibiotic releasing hydroxyapatite, vancomycin-loaded chitosan and antibiotic loaded hydrogels. Due to the increasing problem of antibiotic resistant bacteria, it is necessary to look into non-antibiotic prevention and treatment strategies to prevent from biofilm formation with subsequent PJIs or chronic osteomyelitis. Development focus for PJIs should be on primary prevention of biofilm formation as well on secondary prevention of biofilm formation by preventing bacterial adhesion to an implant surface. In prevention of PJIs coating of implants with e.g., silver nanoparticles or silver ions (Brennan et al., 2015), the inorganic antimicrobial Novaran (Tamai et al., 2009) or antimicrobial peptides current developments (Garrigos et al., 2012; Zhao et al., 2015), which are tested in preclinical stages with promising results.

**TABLE 4:** Examples of current concepts in development of new local antibacterial strategies in prevention of orthopedic infection.

**Types**	**Basic principles**	**Examples**
Passive surface modification	Adhesion/infection preventing surface adjustments	Nano-patterned surface
	Adhesion/infection preventing surface coatings	Anti-adhesive/contact killing polymers
Active surface modification	Inorganic surface leaching coatings	Silver ions/Silver nanoparticles, Novaran
	Organic surface leaching coatings	Antibacterial peptides an polymers, antibiotic loaded hydroxyapatite
Local antibiotic carriers	Biodegradable carriers	Antibiotic containing hydrogels or chitosan
	Non-biodegradable carriers	Antibiotic loaded PMMA

In treatment of chronic osteomyelitis there is a tremendous increase in different biomaterials that might be suitable for local antibiotic delivery. In addition to local antibiotic delivery, these new biomaterials must have good bone defect filing capacities and should be biodegradable. Using these biodegradable local-antibiotic carriers enables the possibility for one-stage treatment of chronic osteomyelitis, which results in lower patient burden, less hospitalization and lower costs. In order to enable on-stage surgery these biomaterials must have proper biocompatibility to stimulate new bone formation and the must not have any toxic side effects on the surrounding tissues. Thereby they should have good biomechanical properties in order to prevent fractures or other mechanical failures after surgery. In the development of these biodegradable antibiotic-loaded biomaterials, different materials have been tested in pre-clinical and clinical studies. Examples of these materials are natural polymers like collagen and chitosan; synthetic polymers like polylactide; ceramics like calcium sulfates and calcium phosphates (hydroxyapatite and tricalcium phosphate) and bioactive glasses (El-Husseiny et al., 2011; Kluin et al., 2013). A recent systematic review regarding treatment of chronic osteomyelitis with antibiotic loaded collagen fleeces or sponges pointed out that good evidence for using these materials is missing (van Vugt et al., 2018). In addition, some other reviews showed that antibiotic-loaded calcium phosphate and calcium sulfate based biomaterials might give better results in treatment of chronic osteomyelitis (McNally et al., 2016; van Vugt et al., 2016). Some of the clinical available antibiotic-loaded biodegradable biomaterials are used with good results, but when looking for new antimicrobial biomaterials problems like antibiotic resistance have to be attacked as well. S53P4 bioactive glass is such a promising non-antibiotic biomaterial able to treat osteomyelitis in a one-stage treatment with good clinical results (Lindfors et al., 2017). It must be taken into account that the evidence for treatment of chronic osteomyelitis with these biomaterials might be further substantiated with additional high quality studies for proper evidence-based decision-making.

## Author Contributions

TvV did the literature research, wrote the first draft, and finalized it to submitted version. JA and JG assisted with literature research, provided additional information, and reviewed the different versions.

## Conflict of Interest Statement

The authors declare that the research was conducted in the absence of any commercial or financial relationships that could be construed as a potential conflict of interest.
